# BrPIF4/BrBBX24-BrHB52-mediated hypocotyl modularity unlocks mechanized harvesting potential in *Brassica rapa*

**DOI:** 10.1093/hr/uhaf328

**Published:** 2025-12-08

**Authors:** Xiaohong Yuan, Yunyun Cao, Xiaoyun Xin, Youping Li, Peirong Li, Weihong Wang, Bin Zhang, Xiuyun Zhao, Yangjun Yu, Deshuang Zhang, Fenglan Zhang, Huarui Wu, Shuancang Yu, Tongbing Su

**Affiliations:** State Key Laboratory of Vegetable Biobreeding, Beijing Vegetable Research Center, Beijing Academy of Agriculture and Forestry Science, Beijing 100097, China; National Engineering Research Center for Vegetables, Beijing Vegetable Research Center, Beijing Academy of Agriculture and Forestry Science, Beijing 100097, China; Beijing Key Laboratory of Crop Molecular Design and Intelligent Breeding, Beijing 100097, China; Key Laboratory of Biology and Genetics Improvement of Horticultural Crops (North China), Beijing 100097, China; State Key Laboratory of Vegetable Biobreeding, Beijing Vegetable Research Center, Beijing Academy of Agriculture and Forestry Science, Beijing 100097, China; National Engineering Research Center for Vegetables, Beijing Vegetable Research Center, Beijing Academy of Agriculture and Forestry Science, Beijing 100097, China; Beijing Key Laboratory of Crop Molecular Design and Intelligent Breeding, Beijing 100097, China; Key Laboratory of Biology and Genetics Improvement of Horticultural Crops (North China), Beijing 100097, China; College of Horticulture Science and Engineering, Shandong Agricultural University, Tai'an 271018, China; State Key Laboratory of Vegetable Biobreeding, Beijing Vegetable Research Center, Beijing Academy of Agriculture and Forestry Science, Beijing 100097, China; National Engineering Research Center for Vegetables, Beijing Vegetable Research Center, Beijing Academy of Agriculture and Forestry Science, Beijing 100097, China; Beijing Key Laboratory of Crop Molecular Design and Intelligent Breeding, Beijing 100097, China; Key Laboratory of Biology and Genetics Improvement of Horticultural Crops (North China), Beijing 100097, China; State Key Laboratory of Vegetable Biobreeding, Beijing Vegetable Research Center, Beijing Academy of Agriculture and Forestry Science, Beijing 100097, China; National Engineering Research Center for Vegetables, Beijing Vegetable Research Center, Beijing Academy of Agriculture and Forestry Science, Beijing 100097, China; Beijing Key Laboratory of Crop Molecular Design and Intelligent Breeding, Beijing 100097, China; Key Laboratory of Biology and Genetics Improvement of Horticultural Crops (North China), Beijing 100097, China; Coyote Bioscience Co., Ltd, Beijing 100089, China; State Key Laboratory of Vegetable Biobreeding, Beijing Vegetable Research Center, Beijing Academy of Agriculture and Forestry Science, Beijing 100097, China; National Engineering Research Center for Vegetables, Beijing Vegetable Research Center, Beijing Academy of Agriculture and Forestry Science, Beijing 100097, China; Beijing Key Laboratory of Crop Molecular Design and Intelligent Breeding, Beijing 100097, China; Key Laboratory of Biology and Genetics Improvement of Horticultural Crops (North China), Beijing 100097, China; State Key Laboratory of Vegetable Biobreeding, Beijing Vegetable Research Center, Beijing Academy of Agriculture and Forestry Science, Beijing 100097, China; National Engineering Research Center for Vegetables, Beijing Vegetable Research Center, Beijing Academy of Agriculture and Forestry Science, Beijing 100097, China; Beijing Key Laboratory of Crop Molecular Design and Intelligent Breeding, Beijing 100097, China; Key Laboratory of Biology and Genetics Improvement of Horticultural Crops (North China), Beijing 100097, China; State Key Laboratory of Vegetable Biobreeding, Beijing Vegetable Research Center, Beijing Academy of Agriculture and Forestry Science, Beijing 100097, China; National Engineering Research Center for Vegetables, Beijing Vegetable Research Center, Beijing Academy of Agriculture and Forestry Science, Beijing 100097, China; Beijing Key Laboratory of Crop Molecular Design and Intelligent Breeding, Beijing 100097, China; Key Laboratory of Biology and Genetics Improvement of Horticultural Crops (North China), Beijing 100097, China; State Key Laboratory of Vegetable Biobreeding, Beijing Vegetable Research Center, Beijing Academy of Agriculture and Forestry Science, Beijing 100097, China; National Engineering Research Center for Vegetables, Beijing Vegetable Research Center, Beijing Academy of Agriculture and Forestry Science, Beijing 100097, China; Beijing Key Laboratory of Crop Molecular Design and Intelligent Breeding, Beijing 100097, China; Key Laboratory of Biology and Genetics Improvement of Horticultural Crops (North China), Beijing 100097, China; State Key Laboratory of Vegetable Biobreeding, Beijing Vegetable Research Center, Beijing Academy of Agriculture and Forestry Science, Beijing 100097, China; National Engineering Research Center for Vegetables, Beijing Vegetable Research Center, Beijing Academy of Agriculture and Forestry Science, Beijing 100097, China; Beijing Key Laboratory of Crop Molecular Design and Intelligent Breeding, Beijing 100097, China; Key Laboratory of Biology and Genetics Improvement of Horticultural Crops (North China), Beijing 100097, China; State Key Laboratory of Vegetable Biobreeding, Beijing Vegetable Research Center, Beijing Academy of Agriculture and Forestry Science, Beijing 100097, China; National Engineering Research Center for Vegetables, Beijing Vegetable Research Center, Beijing Academy of Agriculture and Forestry Science, Beijing 100097, China; Beijing Key Laboratory of Crop Molecular Design and Intelligent Breeding, Beijing 100097, China; Key Laboratory of Biology and Genetics Improvement of Horticultural Crops (North China), Beijing 100097, China; State Key Laboratory of Vegetable Biobreeding, Beijing Vegetable Research Center, Beijing Academy of Agriculture and Forestry Science, Beijing 100097, China; National Engineering Research Center for Vegetables, Beijing Vegetable Research Center, Beijing Academy of Agriculture and Forestry Science, Beijing 100097, China; Beijing Key Laboratory of Crop Molecular Design and Intelligent Breeding, Beijing 100097, China; Key Laboratory of Biology and Genetics Improvement of Horticultural Crops (North China), Beijing 100097, China; Research Center of Information Technology, Beijing Academy of Agriculture and Forestry Sciences, Beijing 100097, China; Key Laboratory of Digital Village Technology, Ministry of Agriculture and Rural Affairs, Beijing 100097, China; State Key Laboratory of Vegetable Biobreeding, Beijing Vegetable Research Center, Beijing Academy of Agriculture and Forestry Science, Beijing 100097, China; National Engineering Research Center for Vegetables, Beijing Vegetable Research Center, Beijing Academy of Agriculture and Forestry Science, Beijing 100097, China; Beijing Key Laboratory of Crop Molecular Design and Intelligent Breeding, Beijing 100097, China; Key Laboratory of Biology and Genetics Improvement of Horticultural Crops (North China), Beijing 100097, China; State Key Laboratory of Vegetable Biobreeding, Beijing Vegetable Research Center, Beijing Academy of Agriculture and Forestry Science, Beijing 100097, China; National Engineering Research Center for Vegetables, Beijing Vegetable Research Center, Beijing Academy of Agriculture and Forestry Science, Beijing 100097, China; Beijing Key Laboratory of Crop Molecular Design and Intelligent Breeding, Beijing 100097, China; Key Laboratory of Biology and Genetics Improvement of Horticultural Crops (North China), Beijing 100097, China

## Abstract

Chinese cabbage production faces critical mechanization challenges due to traditional plant architectures that limit mechanical harvesting efficiency. Traditional breeding prioritized short-hypocotyl varieties to prevent damping-off, but long hypocotyls are now critical for mechanical harvesting. We identified BrHB52, an HD-Zip transcription factor, as a key regulator of hypocotyl elongation through quantitative trait locus (QTL) mapping, RNA-seq, and haplotype analysis. *BrHB52* expression was significantly higher in the long-hypocotyl variety R031L than in the short-hypocotyl variety R032S. Overexpression of *BrHB52* in both Chinese cabbage and Arabidopsis led to elongated hypocotyls. The silencing of *BrHB52* in R031L resulted in a reduction of hypocotyl length. Sequence alignment revealed a 251-bp insertion in the *BrHB52* promoter of the long-hypocotyl variety R031L, which introduced the light-responsive GT-1 motifs. The upstream transcription factors Phytochrome-interacting factor4 (PIF4) and B-box zinc finger 24 (BBX24) were identified through yeast one-hybrid screening using the *BrHB52*^*R031L*^ promoter sequence. PIF4 were found to bind to the both *BrHB52*^*R031L*^ and *BrHB52*^*R032S*^ promoters and activate their expression through G-box, while light-induced factor BBX24 only bind to the *BrHB52*^*R031L*^ promoter and activate its expression by light-responsive element GT-1. Our findings elucidate a BrPIF4/BrBBX24-BrHB52 regulatory module that controls plant architecture through hypocotyl elongation. These findings not only provide critical genetic targets for developing mechanization-compatible Chinese cabbage, but also develop transgenic prototypes with elongated hypocotyls, offering practical resources for mechanized breeding.

## Introduction

With the rising labor costs and increasing demand for mechanization, adapting to mechanized harvesting has emerged as one of the desirable traits in vegetable crops. Chinese cabbage (*Brassica campestris* L. ssp. *pekinensis*) has the highest yield among vegetables in China and it is in need of mechanized harvesting [1, 2]. In the past, varieties with short hypocotyls were more popular in order to prevent damping-off during the seedling stage. However, these varieties are not suitable for mechanized harvesting due to their high losses during the process [3]. Therefore, cultivating varieties of Chinese cabbage with long and thick hypocotyls is currently an urgent breeding objective.

*Arabidopsis thaliana* is a well-studied model organism that helps us understand the complex mechanisms involved in hypocotyl elongation. Light plays a pivotal role in the morphogenesis of plant seedlings. In the absence of light, seedlings go through skotomorphogenesis. This stage is marked by a long hypocotyl, an apical hook, and the closed cotyledons [4]. Conversely, when exposed to light, plants experience photomorphogenesis, leading to a short hypocotyl, open cotyledons, and the accumulation of chlorophyll [4]. Phytochrome interacting factors (PIFs) are essential for suppressing photomorphogenesis in darkness [5, 6]. Elongated hypocotyl 5 (HY5) promotes the expression of light-inducible genes and photomorphogenic development [7]. PIFs inhibit the stability of HY5 protein and maintaining the homeostasis of plant morphogenesis [8]. The B-box zinc finger (BBX) proteins are also central to light signal transduction. BBXs can either directly regulate mRNA level of *HY5*, or interacted with HY5 and PIFs to participate in shade avoidance, photomorphogenesis, and photoperiodic pathway [7, 9–13]. Multiple phytohormone signaling pathways play crucial roles in regulating hypocotyl development. Auxin induces the expression of *Small auxin-up RNA* (*SAUR*) family genes in a dose-dependent manner, which progressively activates plasma membrane proton pump (H + -ATPase) activity, leading to apoplastic acidification in hypocotyl epidermal cells. Moderate acidification promotes cell expansion (acid growth theory), whereas excessive acidification inhibits it [14]. The GA signaling pathway promotes hypocotyl elongation in Arabidopsis through DELLA-PIFs [15] model and the DELLA-ABI4-HY5 [16] model and so on. Brassinosteroids (BRs) promote hypocotyl elongation through their positive regulatory factor Brassinazole-Resistant1 (BZR1), which orchestrates a sophisticated transcriptional network to drive cell expansion [17, 18]. Homeodomain-leucine zipper (HD-Zip) family members are unique to plants. Homeobox protein1 (ATHB1) [19] and Homeobox protein2 (ATHB2) [20, 21] promote hypocotyl elongation in Arabidopsis activated by PIF1. Homeobox protein52 in Arabidopsis (ATHB52) has been shown to positively regulate the expression of *Wavy root growth1/2* (*WAG1/2*), thereby influencing the polar transport of auxin ultimately inhibiting primary root elongation [19]. However, the specific role of ATHB52 in hypocotyl development remains unreported.

Compared with the research on hypocotyls in Arabidopsis, current studies on hypocotyl development in Chinese cabbage are primarily focused on cytological and physiological aspects, with limited exploration of molecular regulatory mechanisms. Early physiological studies have revealed that BRs can stimulate cell wall loosening in hypocotyl cells of Chinese cabbage, thereby promoting hypocotyl growth [22]. Wang and Shang concluded that light intensity is the primary environmental factor regulating this process [23]. Jiang *et al*. overexpressed the Arabidopsis heat-tolerant gene *Heat-induced tas1 target2* (*HTT2*) in Chinese cabbage, suggesting that HTT2 can improve the heat tolerance by promoting hypocotyl elongation [24].

In this study, we identified an HD-Zip family member in *Brassica rapa BrHB52* as a regulator of hypocotyl elongation through QTL mapping, RNA sequencing (RNA-seq), and haplotype analysis. The overexpression of *BrHB52* in both Chinese cabbage and Arabidopsis resulted in elongated hypocotyls, indicating that BrHB52 positively regulate hypocotyl elongation. We identified a 251-bp insertion in the *BrHB52* promoter of the long-hypocotyl variety R031L. This insertion allows two transcription factors to regulate *BrHB52* differently: PIF4 activates *BrHB52* in both R031L and R032S [25], while BBX24 specifically enhances *BrHB52* expression only in R031L. This work reveals a light-responsive BrPIF4/BrBBX24-BrHB52 pathway regulating hypocotyl elongation, and by leveraging this pathway, we have generated transgenic germplasm that fulfills the demand for *B. rapa* crops with morphology optimized for high-density planting and mechanized harvesting systems.

## Results

### Light and hormone signals regulate the hypocotyl growth of Chinese cabbage

**Figure 1:**
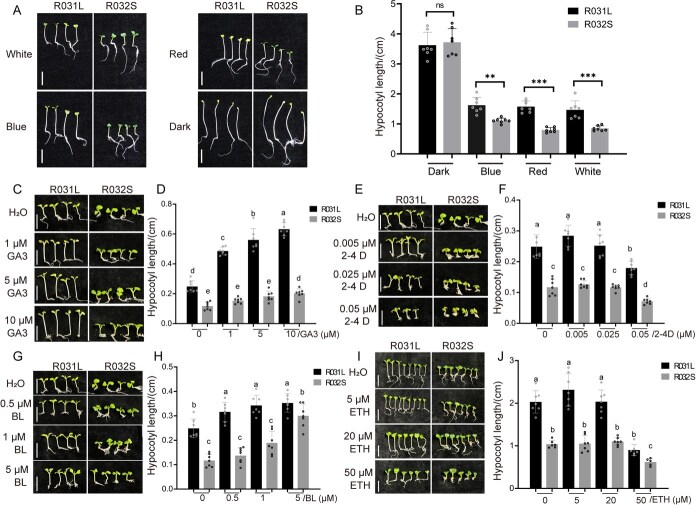
The growth of the hypocotyls in Chinese cabbage varieties R031L and R032S is regulated by both light signals and hormonal signals. (A) Under long-day conditions and in darkness, the hypocotyl characteristics of Chinese cabbage varieties R031L and R032S were observed after 10 days of growth. The plants were exposed to white light, blue light, and red light, each with an intensity of 20 μmol m^−2^ s^−1^. The scale bars on the corresponding images denote 1 cm. (B) The statistical outcomes regarding the length of hypocotyl in (A), along with SEM. Asterisks are used to denote significant disparities between R031L and R032S (^**^P <0 .01, ^***^P <0 .001, Student’s *t*-test). (C) The hypocotyl phenotypes of Chinese cabbage R031L and R032S grown under GA3 treatment. (D) Statistical results of hypocotyl length in C with standard SEM. (E) The hypocotyl phenotypes of Chinese cabbage R031L and R032S grown under 2-4 D treatment. (F) Statistical results of hypocotyl length in E with standard SEM. (G) The hypocotyl phenotypes of Chinese cabbage R031L and R032S grown under BL treatment. (H) Statistical results of hypocotyl length in G with standard SEM. (I) The hypocotyl phenotypes of Chinese cabbage R031L and R032S grown under ETH treatment. (J) Statistical results of hypocotyl length in (I) with standard SEM. Seedlings in (C, E, G, I) were treated for 10 days at white light intensity of 20 μmol m^−2^ s^−1^ under long-day conditions and the scale bars represent 0.5 cm. In (D, F, H, J), identical letters signify nonsignificant disparities, while distinct letters indicate significant differences (*P* <0 .05, two-way analysis of variance (ANOVA)). Data in (B, D, F, H, J) are shown as mean ± SD (*n* = 7 plants). All assays were performed in three independent biological experiments that yielded consistent trends; the Figure displays one representative experiment.

R031L (long-hypocotyl) and R032S (short-hypocotyl) are two Chinese cabbage varieties with contrasting hypocotyl elongation phenotypes. To analyze the regulation of hypocotyl length in R031L and R032S by light signals, the seedlings of Chinese cabbage R031L and R032S were cultured under red, blue, white light, and dark conditions, respectively, and measurements of hypocotyl extension were conducted after 10 days of growth. The results showed that for both R031L and R032S, their hypocotyls were inhibited under blue light, red light, and white light conditions compared with those under dark conditions. Furthermore, there was no obvious difference in hypocotyl length between R031L and R032S etiolated seedlings; while under light conditions, the hypocotyl of R031L was significantly longer than that of R032S (Fig. 1A and B). This result suggested that while light signals generally inhibit hypocotyl elongation in Chinese cabbage, R031L and R032S exhibit distinct light responsiveness, with R031L maintaining longer hypocotyls under light conditions.

R031L and R032S were also treated with various concentrations of gibberellin (GA), auxin, brassinolides (BL), and ethylene (ETH) at white light intensity of 20 μmol m^−2^ s^−1^ under long-day conditions, and the hypocotyl lengths were measured. The results showed that 1, 5, and 10 μM of GA3 all significantly promoted hypocotyl elongation in R031L, with the promotional effect increasing as the GA3 concentration increased. However, the promotional effect of 1 and 5 μM of GA3 on R032S was not significant. When the GA3 concentration reached 10 μM, the hypocotyl of R032S significantly increased in length (Fig. 1C and D). This indicates that the hypocotyl of R031L is more sensitive to GA than R032S. Regarding auxin, 0.005 μM of 2-4D could slightly promote hypocotyl elongation in both R031L and R032S varieties, while 0.025 μM of 2-4D had no significant effect on hypocotyl development. Notably, 0.05 μM of 2-4D significantly inhibited hypocotyl elongation (Fig. 1E and F). Exogenous addition of BL promoted the growth of Chinese cabbage hypocotyls, with R031L more sensitive to lower concentrations (0.5 μM) of BL compared to R032S, but under treatment with higher concentrations (5 μM) of BL, R032S exhibits a greater increase in growth (Fig. 1G and H). Exogenous addition of low concentrations of ethephon had no significant effect on the growth of Chinese cabbage hypocotyls. When the concentration of ethephon reached 50 uM, it significantly inhibited the elongation of Chinese cabbage hypocotyls, with a stronger inhibitory effect on R031L (Fig. 1I and J). These results indicate that GA, auxin, BL, and ethylene are involved in the elongation process of Chinese cabbage hypocotyl, although their effects were different. And differential hypocotyl responsiveness of R031L and R032S to identical phytohormonal treatments reveals underlying divergence in hormone signaling components between different varieties. The above results demonstrate that both light and phytohormonal are involved in the regulation of hypocotyl growth in Chinese cabbage.

### Identification of candidate genes regulating hypocotyl elongation in Chinese cabbage

**Figure 2:**
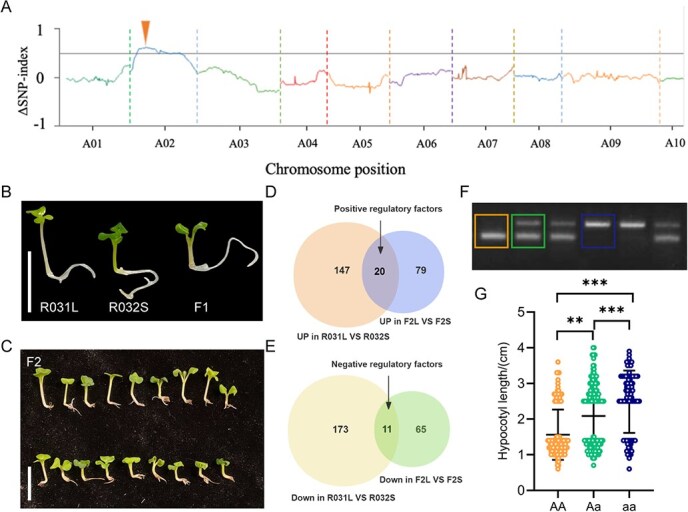
Identification of *BrHB52* as a hypocotyl elongation regulator through integrated QTL mapping, expression, and haplotype analysis. (A) In the F_2_ population resulting from the cross of R031L and R032S, BSA mapping was carried out. The Delta (SNP index) graph generated through BSA-seq analysis pinpointed an interval spanning from 2.6 to 18.6 Mb on chromosome A02. (B, C) Images of the parents and F_1_ generation (B) and some F_2_ individuals (C). Scale bars represent 1 cm. **(**D, E) The Venn diagram represents the number of genes that are upregulated (D) and downregulated (E) in both R031L and F_2_L. (F) The identification results of the *BrHB52* promoter genotype in F_2_ individuals. *n* (total) =371, *n* (AA) = 120, *n* (Aa) = 171, *n* (aa) = 80. (G) Hypocotyl length variation among *BrHB52* promoter haplotypes. (^**^P <0 .01, ^***^P <0 .001, Student’s *t*-test).

The cross between R031L and R032S yielded F_1_ progeny exhibiting a short-hypocotyl phenotype like R032S (Fig. 2B). The segregation of hypocotyl length was observed in the F_2_ generation. Based on the mean and standard deviation (SD) of hypocotyl length measured from 997 F_2_ plants, three phenotypic categories were established: short hypocotyl (*x* ≤ 1.5 cm), intermediate hypocotyl (1.5 cm < *x* < 2.5 cm), and long hypocotyl (*x* ≥ 2.5 cm). In the F_2_ generation, the observed ratio of long hypocotyl, intermediate hypocotyl, and short hypocotyl plants was 265:519:213, consistent with a monogenic incomplete dominance segregation ratio of 1:2:1 (χ^2^ = 3.74, *P* >0 .05). Bulked segregant analysis (BSA) was conducted on an F_2_ population from a cross between the R031L and R032S, selecting extreme phenotypes for pool construction (Fig. 2C). For BSA analysis, we generated high-quality sequencing data from the long-hypocotyl bulk (F_2_L, *n* = 50) and short-hypocotyl bulk (F_2_S, *n* = 50), respectively (F_2_S). In the F_2_L and F_2_S bulks, 291 631 and 291 894 single nucleotide polymorphisms (SNPs) were identified, respectively. The D (SNP index) plot revealed distinct patterns in the SNP index for the F_2_L and F_2_S bulks in the region from 2 615 000 to 18 575 000 bp on chromosome A02 (Fig. 2A). Within this 15.96-Mb interval, there are a total of 2368 genes.

RNA-seq was performed using hypocotyl of 10-day-old seedlings from R031L, R032S, F_2_L, and F_2_S. Transcriptome data analysis revealed that a total of 1615 genes within the interval were expressed in both F_2_L and F_2_S, while 1596 genes were expressed in both R031L and R032S. A total of 144 differentially expressed genes (DEGs) were identified between F_2_L and F_2_S, with 79 genes upregulated and 65 genes downregulated in F_2_L compared to F_2_S. Similarly, 320 DEGs were found between R031L and R032S. Compared to R032S, 147 genes were upregulated and 173 genes were downregulated in R031L. In summary, there were 20 genes upregulated (Fig. 2D) and 11 genes downregulated (Fig. 2E) in both R031L and F_2_L. Among the 20 genes, only homologous gene of HB52 in *A. thaliana* has been previously documented in hormone-mediated growth and development processes [26]. Considering that the hypocotyl length of the combination of R031L and R032S is regulated by hormones such as ethylene and auxin (Fig. 1), we hypothesized that BrHB52 might also participate in hypocotyl development in Chinese cabbage.

To verify this hypothesis, the haplotype analysis was conducted of *BrHB52* in F_2_ individuals with different hypocotyl lengths. In the F_2_ population, the promoter of *BrHB52* exhibits three genotypes: the homozygous genotype with only the longer promoter like R031L (aa), the homozygous genotype with only the shorter promoter like R032S (AA), and the heterozygous genotype Aa (Fig. 2F). We found that there was a significant correlation between the *BrHB52* promoter genotype and hypocotyl length. Specifically, the average hypocotyl length was the longest in plants with a homozygous R031L-type (aa) *BrHB52* promoter, intermediate in heterozygotes (Aa), and shortest in plants with a homozygous R032S-type (AA) *BrHB52* promoter (Fig. 2G). The results suggest that BrHB52 may be involved in the regulation of hypocotyl growth in Chinese cabbage.

### The expression level of *BrHB52* is suppressed by light and correlates with the hypocotyl length

**Figure 3:**
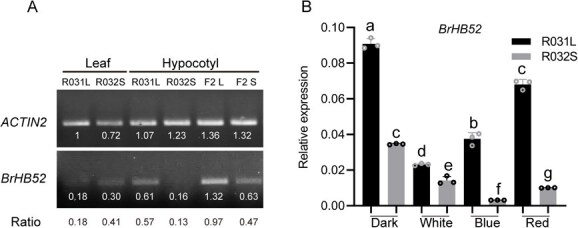
The expression level of *BrHB52* is high in Chinese cabbage with long hypocotyls, and its expression is inhibited by light. (A) Semiquantitative PCR analysis of *BrHB52* expression in Chinese cabbage leaves and hypocotyls with *ACTIN2* as the reference gene. F_2_ L represents the long hypocotyl F_2_ bulk. F_2_ S represents the short hypocotyl F_2_ bulk. The values below the bands represent the grayscale intensities of the bands analyzed by ImageJ software, while the values below the gel image indicate the ratio of the grayscale intensity of the *BrHB52* PCR band to that of the internal reference *actin2* band in the same sample, serving as an indicator of gene transcription level. (B) qRT-PCR analysis of *BrHB52* expression under white light, blue light, red light, and dark treatments, with *ACTIN2* as the reference gene. Extract RNA from hypocotyls of R031L and R032S grown for 10 days at different light with 20 μmol m^−2^ s^−1^ intensity under long-day conditions. The same and different letters represent nonsignificant and significant differences, respectively (*P* <0 .05, two-way ANOVA).

Through semiquantitative expression pattern analysis, it was found that the *BrHB52* expression level in the hypocotyl was significantly higher than that in leaves. Consistent with the results of transcriptome (Fig. S1A), the expression of *BrHB52* in the hypocotyls of R031L is significantly higher than that in R032S, and the expression in F_2_L is higher than that in F_2_S (Fig. 3A).

Since light inhibits the elongation of hypocotyls in both R031L and R032S, and the difference in hypocotyl length between them is more pronounced when grown under light (Fig. 1A and B). Transcription levels of *BrHB52* in the hypocotyl under different light treatments were compared through quantitative real-time polymerase chain reaction (qRT-PCR). The results showed that in etiolated seedlings grown under dark conditions, the *BrHB52* transcriptional level were relatively high in both R031L and R032S. And in hypocotyl of seedlings grown under white, blue, or red light, the expression of *BrHB52* in R031L and R032S were both significantly reduced. This indicated that *BrHB52* transcriptional level in the hypocotyls was suppressed by light (Fig. 3B), as well as the length of hypocotyl (Fig. 1A and B). Besides, whether under light or dark conditions, the expression level of *BrHB52* in R031L hypocotyls was higher than that in R032S (Fig. 3B). These indicated a tight correlation between *BrHB52* expression and hypocotyl length in R031L and R032S, and suggesting a potential positive regulatory role of BrHB52 in hypocotyl elongation in Chinese cabbage.

### BrHB52 is a positive regulator for hypocotyl elongation

**Figure 4:**
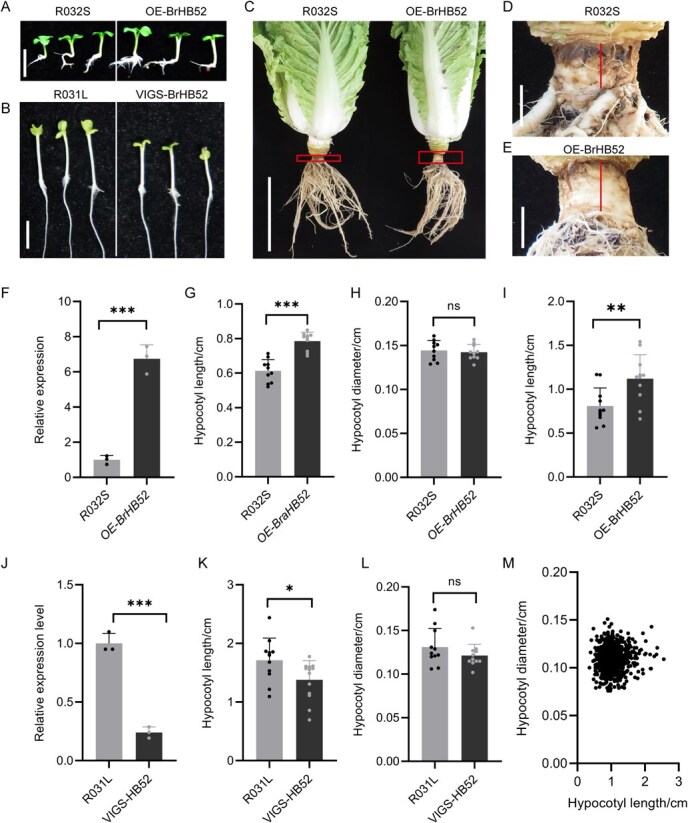
BrHB52 positively regulates hypocotyl elongation in Arabidopsis and Chinese cabbage. (A) *BrHB52* overexpression in R032S increased hypocotyl length grown for 7 days under white light with intensity of 50 μmol m^−2^ s^−1^ in the incubator under long-day conditions. Scale bars represent 1 cm. (B) The silencing of *BrHB52* in R031L resulted in a reduction of hypocotyl length when grown for 7 days under white light at an intensity of 50 μmol m^−2^ s^−1^ in an incubator with long-day conditions. Scale bars represent 1 cm. (C) Overall view of *BrHB52* overexpression lines and R032S at 90 days after sowing. Scale bars represent 10 cm. (D, E) Image of the hypocotyl from 90-day-old R032S (D) and *BrHB52* overexpression lines (E). Scale bars represent 1 cm. (F) The expression of *BrHB52* in Chinese cabbage R032S and transgenic plants *OE-BrHB52*. Asterisks are used to denote significant disparities between R032S and the transgenic plants (^***^P <0 .001, Student’s *t*-test). (G) Statistical results of hypocotyl length in R032S and *OE-BrHB52* for 7 days under white light with intensity of 50 μmol m^−2^ s^−1^ in the incubator under long-day conditions. Asterisks are used to denote significant disparities between R032S and the transgenic plants (^***^P <0 .001, Student’s *t*-test, *n* = 10). (H) Statistical results of hypocotyl thickness in R032S and *OE-BrHB52* for 7 days (‘ns’ denotes the absence of a significant disparity, as determined by Student’s *t*-test, *n* = 10) (I) Statistical results of hypocotyl length in R032S and *OE-BrHB52* for 90 days. Asterisks are used to denote significant disparities between R032S and the transgenic plants (**P <0 .01, Student’s *t*-test, *n* = 10). (J) The expression of *BrHB52* in Chinese cabbage R032S and *VIGS-BraHB52*. Asterisks are used to denote significant disparities between R032S and the transgenic plants (^***^P <0 .001, Student’s *t*-test). (K) Statistical results of hypocotyl length in R032S and *VIGS-BraHB52*. Asterisks are used to denote significant disparities between R032S and the transgenic plants (^*^P <0 .05, Student’s *t*-test, *n* = 10). (L) Statistical results of hypocotyl thickness in R031L and *VIGS-BraHB52.* The notation ‘ns’ denotes that there is no significant disparity, as determined by the Student’s *t*-test, *n* = 10. (M) Regression analysis of hypocotyl length and diameter in F_2_ seedlings derived from R031L and R032S, *R^2^* = 0.0006.

To elucidate the functional role of BrHB52 in controlling hypocotyl growth, the *35S:BrHB52* constructs were transformed into short-hypocotyl Chinese cabbage R032S, generating *BrHB52* overexpression lines (*OE-BrHB52*). Whether it is the 7-day-old seedlings (Fig. 4A and G) or the mature cabbage plants for 90 days (Fig. 4C–E, I), the hypocotyls of *OE-BrHB52* were significantly longer than those of the R032S. The qRT-PCR demonstrated that the transcriptional of *BrHB52* were upregulated by 6-fold in *OE-BrHB52* compared to the R032S (Fig. 4F). Furthermore, the virus-induced gene silencing (VIGS) system were used to treat the exposed seeds, silencing *BrHB52* in the long-hypocotyl material R031L. *BrHB52* expression was reduced to about one-fifth of that of the R031L (Fig. 4J) and silencing of *BrHB52* effectively shortened the length of the hypocotyl (Fig. 4B and K). The overexpression lines of *BrHB52* in Arabidopsis were also generated. Upon observation under short-day conditions for 9 days, we noted that the hypocotyls of Arabidopsis plants overexpressing *BrHB52* were elongated compared to the wild-type control (Fig. S2A and D), while there was no obvious difference under long-day conditions (Fig. S2B and E). To our surprise, functional divergence analysis revealed that *AtHB52*, the Arabidopsis ortholog of *BrHB52*, does not contribute to hypocotyl development, as *athb52* mutants and *OE-AtHB52* overexpression lines showed comparable hypocotyl lengths to wild-type Col-0 (Fig. S2C, F–G).

In addition, to explore whether BrHB52 affects the thickness of the hypocotyl while promoting its elongation of Chinese cabbage, the hypocotyl diameters of *OE-BrHB52 and VIGS-HB52* were measured and it showed that there was no obvious change in the thickness of the hypocotyls of them (Fig. 4H and L). We also conducted a regression analysis on the length and thickness of the hypocotyls of F_2_ individuals, there was no significant correlation between the length and thickness (Fig. 4M). In summary, these indicate that BrHB52 exerts a positive regulatory effect on hypocotyl elongation in both Arabidopsis and Chinese cabbage, while having no impact on hypocotyl thickness.

### *BrHB52* is a direct target of BrPIF4 and BrBBX24

**Figure 5:**
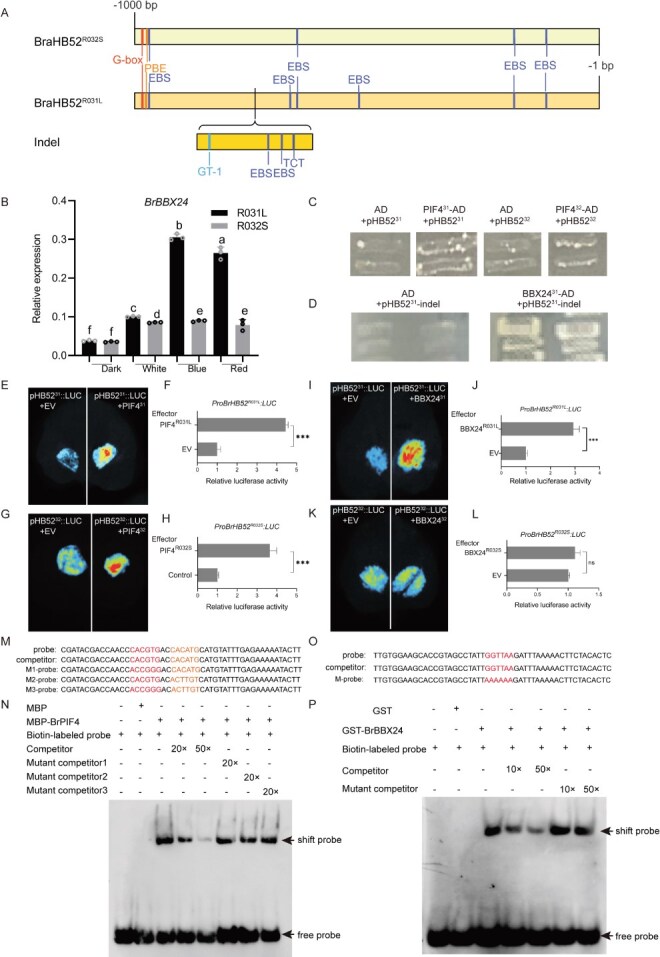
BrPIF4 and BrBBX24 bind to and activate the *BrHB52* promoter. (A) Schematic diagram of promoter sequence alignment between *BrHB52* from R031L and R032S. (B) qRT-PCR analysis of *BrBBX24* expression under light and dark treatments, with *ACTIN2* as the reference gene. Extract RNA from hypocotyls of R031L and R032S grown for 10 days under different light with 20 μmol m^−2^ s^−1^ intensity under long-day conditions. The same and different letters represent nonsignificant and significant differences, respectively (*P* <0 .05, two-way ANOVA). (C) Y1H assays of BrPIF4^R031L^ to the *BrHB52^R031L^* promoter and BrPIF4^R032S^ to the *BrHB52^R032S^* promoter. AD empty vector with pABAi-pBrHB52*^R031L^* and pABAi-pBrHB52*^R032S^* plasmid were cotransformed into Y1H Gold yeast cells as the negative control, respectively. (D) Yeast transcriptional activation assays of BrBBX24^R031L^ to the 251-bp insertion fragment in the promoter *BrHB52^R031L^*. AD empty vector and pABAi-pBrHB52 *^R031L^* and plasmid were cotransformed as the negative control. (E) Dual luciferase reporter assay demonstrated that BrPIF4^R031L^ activate *BrHB52^R031L^*. (F) The relative activity of luciferase of (E) (^***^P <0 .001, Student’s *t*-test). (G) Dual luciferase reporter assay demonstrated that BrPIF4^R032S^ activate *BrHB52^R032S^*. (H) The relative activity of luciferase of (G) (^***^P <0 .001, Student’s *t*-test). (I) Dual luciferase reporter assay demonstrated that BrBBX24^R031L^ activate *BrBBX24^R031L^*. (J) The relative activity of luciferase of (I) (^***^P <0 .001, Student’s *t*-test). (K) Dual luciferase reporter assay demonstrated that BrBBX24^R032S^ activate *BrBBX24^R032S^*. (L) The relative activity of luciferase of K (‘ns’ denotes the absence of a significant disparity, as determined by Student’s *t*-test.) (M) The probes used in EMSA for BrPIF4 binding G-box. A 5′-biotinylated oligonucleotide was used as the probes. The unlabeled probe with the same sequence as the labeled probe serves as the competitive probe. The competitive probe with the G-box (CACGTG) mutated is designated as the competitive mutant probe 1. The competitive probe with the E-box (CACATG) mutated is the competitive mutant probe 2. The competitive probe with both the G-box and E-box mutated is the competitive mutant probe 3. (N) EMSA for BrPIF4 binding G-box in the *BrHB52* promoter. (O) The probes used in EMSA for BrBBX24-binding CT-1 motif. A 5′-biotinylated oligonucleotide was used as the probes. The unlabeled probe serves as the competitive probe. The competitive probe with the GT-1 element (GGTTAA) mutated is designated as the competitive mutant probe. (P) EMSA for BrBBX24 binding GT-1 motif in the *BrHB52* promoter.

In order to investigate the reasons for the difference in *BrHB52* expression in the hypocotyls of R031L and R032S. The promoter sequences of *BrHB52* were cloned from R031L and R032S, respectively. *BrHB52* exhibits pronounced differences in promoter regions between R031L and R032S. The promoter region contains a number of SNPs, and additionally, there is a 251-bp fragment insertion specifically in the promoter of R031L that is not present in R032S. There are four potential EIN3 binding sites (EBS; TACAT) and two light-responsive elements, G-box (CACGTG) and PBE-box (CACATG) on the same sequence segment of the *BrHB52* promoters from R031L and R032S (Fig. 5A and Fig. S1C). The 251-bp insertion introduces additional two EBS and two light-responsive elements: the GT1-motif (CCAATT) and the TCT motif (TCTTAC). To investigate the relationship between these elements and the differential expression of *BrHB52* in R031 and R032, the transcriptional level of *BrHB52* in R031 and R032 after ethylene treatment was determined firstly. The results showed that there was no significant difference in the effect of ethylene on the BrHB52 expression in R031 and R032 (Fig. S1B). We therefore focused our attention on the two light-responsive elements.

To uncover the upstream regulators that modulate the expression of *BrHB52*, yeast one-hybrid (Y1H) screening experiments were conducted utilizing the *BrHB52^R031L^* promoter as the bait. The PIF4 was shown to be inhibited by light signals and are positive regulators of hypocotyl growth [27]. The results of Y1H (Fig. 5C) and dual luciferase reporter assay showed that BrPIF4 could bind to and activate both the promoter of *BrHB52^R031L^* (Fig. 5E and F) and *BrHB52^R032S^* (Fig. 5G and H). The electrophoretic mobility shift assays (EMSA) demonstrated that BrPIF4 could bind to the G-box and E-box on the *BrHB52* promoter (Fig. 5M and N). Additionally, Y1H screening also identified the B-box zinc finger 24 (BBX24). *BBX24* is induced by light and positively regulates hypocotyl development in Arabidopsis [28]. The expression of *BrBBX24* is also induced by light in Chinese cabbage (Fig. 5B). BBX24 has been reported to bind to the GT-1 element of the promoters of *peroxidases 17* (*PRX17*) [29]. In the inserted fragment of the *BrHB52^R031L^* promoter, an additional light-responsive element GT-1 was added. The Y1H (Fig. 5D), dual luciferase reporter assay (Fig. 5I–L) and EMSA (Fig. 5O and P) showed that BBX24 could bind to the GT-1 element on the *BrHB52^R031L^* promoter and activate the expression of *BrHB52^R031L^*. These indicate that light-suppressed BrPIF4 directly activate the expression of *BrHB52* from R031L and R032S, while light-induced BrBBX24 directly only activate the expression of *BrHB52* from R031L, which partially explains the difference in expression levels of *BrHB52* between R031L and R032S under dark and light conditions.

## Discussion

### BrHB52 promotes hypocotyl elongation downstream of BrPIF4 and BrBBX24

**Figure 6:**
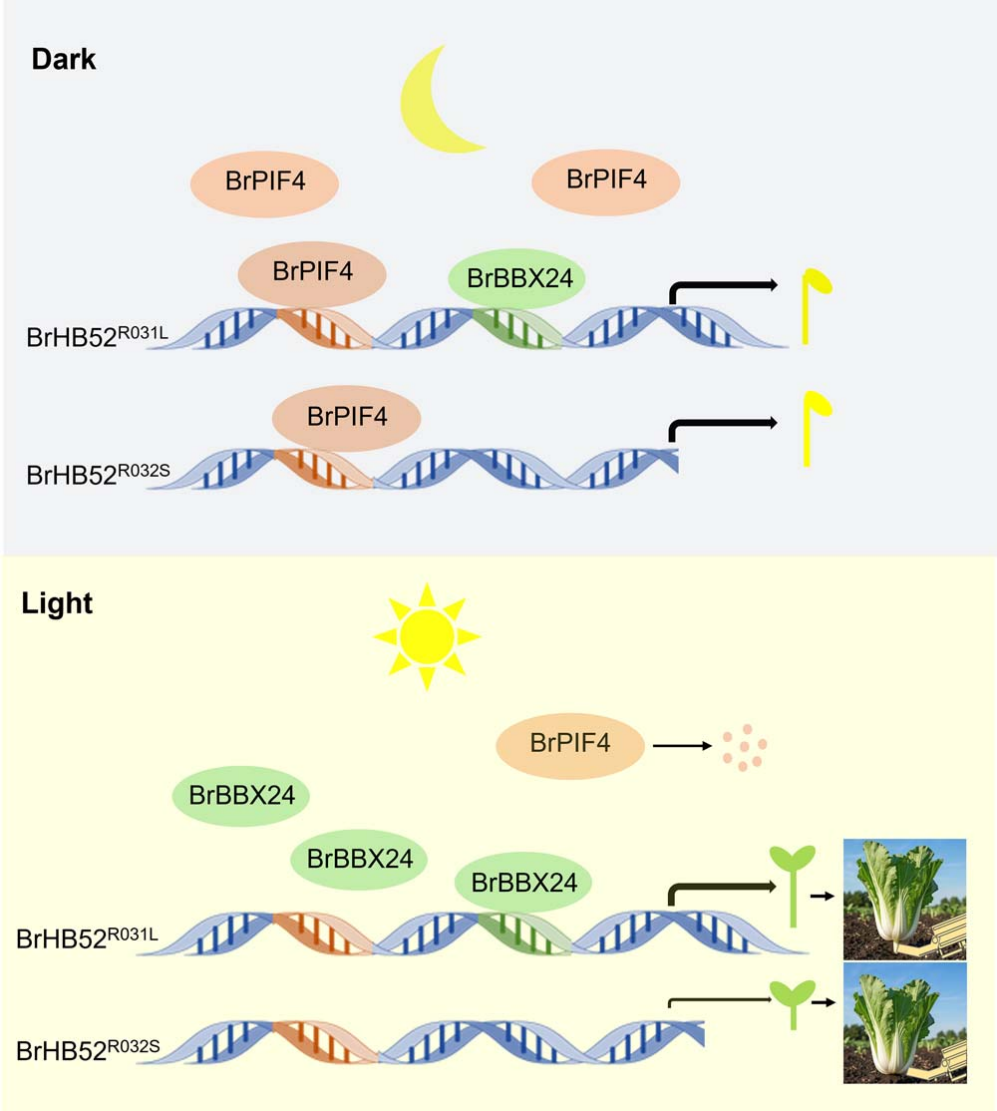
Regulatory model of BrHB52-mediated hypocotyl elongation. In long-hypocotyl Chinese cabbage (R031L), BrPIF4 activates BrHB52 by binding the conserved G-box promoter motif under darkness, while light induces BrBBX24 to specifically bind the R031L-unique 251bp insertion containing GT-1 elements, synergistically enhancing BrHB52 expression; conversely, short-hypocotyl plant (R032S) lacks this insertion, preventing BrBBX24 binding and resulting in light-triggered BrPIF4 degradation that suppresses BrHB52 expression.

ATHBs in Arabidopsis regulate biological processes such as photomorphogenesis, flower development, and plant responses to stress by interacting with other proteins and participating in hormone-mediated signaling pathways [30]. Through QTL mapping in an F_2_ population combined with RNA-seq and haplotype analysis, we identified the HD-Zip member BrHB52, which regulates the hypocotyl of Chinese cabbage (Fig. 2). Furthermore, BrHB52 promotes hypocotyl elongation in both Arabidopsis and Chinese cabbage (Fig. 4). BrPIF4 can bind to the G-box element on the *BrHB52* promoter and activate the expression of *BrHB52* both in R031L and R032S. But the effect on *BrHB52^R031L^* is stronger than that on *BrHB52^R032S^* (Fig. 6E–H). Both the promoters of *BrHB52^R031L^* and *BrHB52^R032S^* contain one G-box site, but PIF4 binds to and activates *BrHB52^R031L^* more strongly than *BrHB52^R032S^*, possibly due to the presence of G-box coupling elements (GCEs) ACGC in the insert fragment of *BrHB52^R031L^*. According to reports, GCEs can be bound by interacting proteins of PIF, thereby stabilizing PIF’s binding to the binding element [31].

Based on the results, we speculated such a working model (Fig. 6). In darkness, *BrHB52^R031L^* and *BrHB52^R032S^* are both activated by BrPIF4, leading to relatively high expression levels that promote hypocotyl elongation. Since BrPIF4 activates *BrHB52^R031L^* more strongly than *BrHB52^R032S^*, the *BrHB52^R031L^* expression is higher than that of *BrHB52^R032S^* in darkness. Under light conditions, BrPIF4 undergo degradation, while the expression of *BrBBX24* increases in response to light induction. BrBBX24 activates the expression of *BrHB52^R031L^* by binding to GT-1 motif but not *BrHB52^R032S^* under light conditions. Therefore, the expression level of *BrHB52^R032S^* becomes very low under light conditions.

### The development of the hypocotyl in Chinese cabbage

Studies on hypocotyl elongation in *A. thaliana* have uncovered a complex genetic regulatory network that is finely tuned by signals from light, hormones, and temperature [32]. Although the length of the hypocotyl is crucial to the efficiency of mechanized harvesting of Chinese cabbage, there are currently still relatively few studies on the development of the hypocotyl in Chinese cabbage. In this study, we observed that light inhibited the growth of the hypocotyl of Chinese cabbage, just as in the studies of other plants [33].

While regarding the impact of phytohormone on the hypocotyl of Chinese cabbage, we found that GA stimulates hypocotyl elongation in Chinese cabbage (Fig. 1C and D), also aligning with findings in Arabidopsis [34].

Although auxin generally stimulates hypocotyl elongation, an overabundance of auxin inhibits this process in Arabidopsis [14]. We observed that low concentrations of auxin could slightly promote hypocotyl elongation in both R031L and R032S varieties, whereas high concentrations of auxin significantly inhibited hypocotyl elongation (Fig. 1E and F). These findings are generally consistent with the role of auxin in regulating Arabidopsis hypocotyl elongation. This also suggests that for both R031L and R032S varieties of Chinese cabbage, the endogenous auxin content in their hypocotyls is close to the optimal concentration for elongation, additional auxin application may even inhibit their hypocotyl elongation. Furthermore, the effect of auxin on hypocotyls is also related to light signal, as promoting growth in light and inhibiting elongation in darkness [35–37]. However, in this study, the influence of auxin on the hypocotyl growth of Chinese cabbage was only observed under white light (20 μmol m^−2^ s^−1^, long-day photoperiod), limiting our understanding of auxin–hypocotyl interactions in darkness or other light spectra (e.g. red/blue light). Therefore, more experiments may be needed for further analysis in the future.

BRs have the effects of promoting plant growth and cell expansion in Arabidopsis [38, 39]. In this study, BR also promotes hypocotyl elongation in both R031L and R032S. Besides, when treated with 5 μM BL, the hypocotyl length of R032S increased to the level of untreated R031L (Fig. 1G and H), suggesting that the endogenous BR content in the hypocotyl of R031L may be higher than that in R032S.

Ethylene promotes hypocotyl elongation grown in light but strongly suppresses it in etiolated (dark-grown) seedlings in Arabidopsis [40, 41]. In this study, the effect of ethylene on the hypocotyl of Chinese cabbage was observed under weak white light conditions. The results showed that low concentrations of ethylene hardly affected the hypocotyl length, while high concentrations inhibited hypocotyl elongation (Fig. 1I and J). This is somewhat different from the conclusions drawn in Arabidopsis. However, the mechanism behind it remains unknown, and there have been no relevant reports in Chinese cabbage to date.

We also observed and measured the hypocotyl lengths of *athb52* and *OE-ATHB52*, and found no significant differences compared to the Col-0 (Fig. S2C and G). BrHB52 appears to be species-specifically regulated relative to AtHB52. Although the proteins share ~85% identity (Fig. S3), BrHB52 contains an additional 33 amino acids at the N-terminus and lacks 20 amino acids at the C-terminus compared with AtHB52, which may alter conformation, interactions, or activity. Expression also diverges: AtHB52 is root-biased with no detectable signal in 10-day hypocotyls [26], whereas BrHB52 shows robust hypocotyl expression in 10-day R031L seedlings (~0.6× ACTIN; Fig. 3A). There are also differences in flowering mechanisms between Arabidopsis and cabbage [42]: both *braA.ref6* and *atref6* flower late, yet REF6 acts via GA metabolism in Chinese cabbage and via FLC repression in Arabidopsis [43]; heat delays flowering in Chinese cabbage by increasing H2A.Z at BrFT, while it accelerates flowering in Arabidopsis by lowering H2A.Z at FT [44]. These patterns support lineage-specific regulatory contexts for HB52-like factors and align with the dual light/dark control of BrHB52 described here.

## Materials and methods

### Plant material and growth conditions

Seeds of Col-0 and transgenic plants underwent sterilization. Subsequently, they were kept at 4°C for 4 days. After that, the seeds were sown on half-strength Murashige and Skoog (MS) medium. Once the seedlings reached 8 days old, they were moved to soil within a growth chamber. The growth chamber was maintained at 22°C. Seeds from inbred lines or transgenic specimens of Chinese cabbage were allowed to sprout at room temperature over a 2-day period. Subsequently, these germinated seeds were transplanted into soil within a greenhouse with 21°C–28°C (light phase) and 12°C–20°C (dark phase).

For the assessment of hypocotyl length in Chinese cabbage plants, they were arranged in an eight-row by five-column layout within a petri dish. This dish was filled with 4 ml of water and lined with three layers of absorbent paper. The petri dish was then placed inside an incubator for cultivation. The specific photoperiod, light composition, and light intensity were set according to the specifications of each experiment, and these details are provided in the figure legend. In the hormone treatment experiment, the deionized water in the setup was substituted with a hormone solution.

Regarding phenotypic observations of mature Chinese cabbage, we transplanted *OE-BrHB52* and R032S to Zhangjiakou (ZJK, 114:55E/40:51 N).

### Virus induced gene silencing assays

For the seed VIGS assay, a slightly modified version of the protocol described by [45] was utilized. A 300-bp sequence from the coding DNA sequences (CDS) of *BrHB52* was cloned and integrated into the PTRV2 vector (HG-VRW0366, HonorGene, Hunan, China), generating the PTRV2-HB52 construct. Subsequently, this construct was introduced into Agrobacterium (GV3101). Germinating R031L seeds were immersed in Agrobacterium cultures (with an OD_600_ value of 1.5). The culture was a 1:1 combination of PTRV1 (HG-VRW0365, HonorGene, Hunan, China) and PTRV2-HB52. As a control, seeds were soaked in an Agrobacterium solution where PTRV1 and PTRV2 were mixed. Once the seeds were subjected to Agrobacterium infection, following 1 day of imbibition during the infection process, the treated seeds were moved to a culture medium having 0.8% plant gel. These seeds were then incubated under long-day conditions in an incubator. They were exposed to white light at an intensity of 50 μmol m^−2^ s^−1^ for a period of 7 days.

### RNA, DNA extraction, and polymerase chain reaction

The total DNA was extracted from leaves by means of the cetyltrimethylammonium bromide (CTAB) approach. Total RNA isolation was performed using a kit (DP441, Tiangen, Beijing, China). After that, the RNA underwent reverse transcription by Hiscript III Reverse Transcriptase (R302–01, Vazyme, Nanjing, China). qRT-PCR assays were carried out on a Roche thermocycler (LightCycler 480, Roche, Basel, Switzerland). Transcript levels were measured using relative quantification as detailed by Pfaffl [46], with the actin gene of Chinese cabbage acting as the internal control. The semiquantitative PCR in Fig. 3 was performed using 2 × Rapid Taq Master Mix (P222, Vazyme, Nanjing, China) with the system as: Template cDNA (200 ng), 10 μM forward and reverse primers (1 μl), 2 × Rapid Taq Master Mix (1 μl), and ddH₂O (to a total volume of 20 μl). The protocol is as follows: initial denaturation at 95°C for 3 min, followed by 30 cycles of 95°C for 15 s (denaturation), 58°C for 15 s (annealing), and 72°C for 15 s (extension), with a final extension at 72°C for 5 min. The primers in the semiquantitative PCR were: RT-BrHB52-F: CTGCAATCTCCAGTCCAAGC; RT-BrHB52-R: AAGCTCGTCAAAGACCACCA; RT-Actin-F: CAGGTTTGGAATTGTCGAGG; RT-Actin-R: GAGCTGTGGAAGCACCTTTC.

### RNA sequencing assays

In the context of RNA-seq, we obtained RNA from the hypocotyls of 10-day-old parental strains (R031L and R032S) as well as the F_2_ bulk. These samples were cultivated under long-day white light conditions, where the light intensity was set at 20 μmol·m^−2^·s^−1^.

Total RNA isolation was performed using a kit (DP441, Tiangen, Beijing, China). The sequencing of cDNA library was by Illumina (San Diego, CA). The reference genome was from the Brassicaceae Database (BRAD, http://www.brassicadb.cn/#/). HISAT2 software was used for alignment task. StringTie software was used for assembly in a reference-dependent manner. The significant DEGs were set as A Fold Change ≥2 and *P*-value ≤ .5. KOBAS package was employed for Kyoto Encyclopedia of Genes and Genomes (KEGG) and Gene Ontology (GO) analysis.

### BSA analyses and QTL mapping

For the analysis of BSA, we obtained DNA from the hypocotyls of 10-day-old parental lines, namely R031L and R032S, as well as from the F_2_ population. To prepare the two DNA bulks for BSA sequencing, we selected individuals from the F_2_ population. Bulk ‘F_2_L’ was created by combining equal quantities of DNA from 50 individuals that exhibited the long-hypocotyl characteristic. Conversely, bulk ‘F_2_S’ was formed by DNA from 50 individuals showing the short-hypocotyl trait [47]. The bulk segregation analyses were carried out by Majorbio Company (Beijing, China). The experimental approach was based on the method described in [47].

### Yeast one-hybrid assay

Using the seamless cloning approach with restriction enzymes *EcoR*I and *Sac*I, the CDS of *BrPIF4* and *BrBBX24* from R031L and R032S were inserted into the pGADT7 vector. Similarly, the promoters of *BrHB52* from R031L and R032S were cloned into the pAbAi vector via seamless cloning, using the restriction enzymes *Kpn*I and *Xho*I. The relevant primers are detailed in Table S1. The experimental procedures were carried out according to the instructions of the kit (YH1001-10 T, Coolaber, Beijing, China).

### Dual luciferase reporter assay

The CDS of *BrPIF4* and *BrBBX24* were cloned and then inserted into the pCAMBIA2300 vector to create effectors. This was achieved through seamless cloning, followed by digestion with the restriction enzymes *Kpn*I and *Sal*I. Meanwhile, the promoter of *BrHB52* was inserted into the pGreenII0800 vectors by *Hin*dIII and *Bam*HI. After constructing the effectors and reporters, they were coinjected into the epidermal cells of tobacco then incubated for a period of 48 h. As a control, pCAMBIA2300 and pGreen-HB52 were coinjected. To measure the Firefly Luciferase activities, the FUSION FX EDGE/DBT (VILBER, China) system was utilized. The quantitative determination of relative enzyme activity was performed using the Dual Luciferase Reporter Gene Assay Kit (11402ES60, Yeasen, Shanghai, China) according to the manufacturer’s instructions.

### Electrophoretic mobility shift assays

The CDS of *BrPIF4* was integrated into the pMAL-c2x vector carrying a maltose-binding protein (MBP) tag. Biotinylated oligonucleotides labeled at the 5′ end were employed as probes. An unlabeled oligonucleotide as the labeled probe functioned as a competitive probe. When the G-box (CACGTG) within the competitive probe was altered, the resulting probe was named competitive mutant probe 1. If the E-box (CACATG) in the competitive probe was mutated, it was referred to as competitive mutant probe 2. In the case where both the two box in the competitive probe were mutated, it became competitive mutant probe 3.

The CDS of *BrBBX24* was cloned into the pGEX-6P-1 vector tagged with glutathione S-transferase (GST). 5′-biotinylated oligonucleotides were used as probes. The unlabeled probe with an identical sequence to the labeled one acted as a competitive probe. When the GT-1 element (GGTTAA) in the competitive probe was mutated, it was labeled as the competitive mutant probe. The specific sequences of the probes are presented in Table S1. An EMSA was conducted using the LightShift Chemiluminescent EMSA Kit (Thermo Scientific, Waltham, USA).

### Construction of OE-BrHB52 vector and genetic transformation

For the construction of the *BrHB52* overexpression vector, the CDS of *BrHB52* from R031L was cloned and then inserted into the pCAMBIA2300 vector to create pCAMBIA2300-BrHB52 vector**.** This was achieved through seamless cloning, followed by digestion with the restriction enzymes *Kpn*I and *Sal*I. The primers used for vector construction are listed in Table S1. The constructed plasmid was transformed into *Agrobacterium tumefaciens* strain GV3101. The method of genetic transformation in Chinese cabbage was referred to Xin *et al* [48]. Single colonies of Agrobacterium were cultured for 48 h, then centrifuged and resuspended by liquid MS to an OD_600_ of 0.5. Seeds were sterilized and then placed on germination medium at 25°C for 4 days. Cutten 1- to 2-mm cotyledonary petioles of 4-day-old seedlings were excised and placed on callus induction media for cocultivation (CIM-C) medium. The ends of the petioles were immersed in the Agrobacterium suspension for treatment. The explants were transferred back to the same CIM-C plates and cultured in a dark chamber at 25°C for 72 h. They were then placed on shoot induction media (SIM) medium for 2 weeks, followed by transfer to fresh SIM medium until shoots emerged. Subsequently, they were transferred to shoot induction media (SEM) medium for growth for 1–2 weeks, and then to rooting medium (RM) for growth for 1–3 weeks. After hardening the roots in the growth chamber for 2 days, the plants were transplanted into soil.

Plant culture media are listed as below:

Liquid MS: 4.3 g/l MS basal salts (M519, Phytotech, USA), 30 g/l Sucrose, pH 5.7; Germination media (GM): 4.3 g/l MS basal salts, 30 g/l Sucrose, pH 5.7, and 8 g/l agar; CIM-C: GM plus 500 mg/l 2-(N-morpholino) ethanesulfonic acid (MES), 4 mg/l 6-BA, and 0.667 mg/l IAA; SIM: CIM-C medium plus 160 mg/l Timentin, 2 mg/l AgNO3, and 25 mg/l kanamycin; SEM: GM plus 500 mg/l MES, 160 mg/l Timentin, 50 μg/l 6-BA, 2 mg/l AgNO3, and 25 mg/l kanamycin; RM: 3.05 g/l Gamborg’s B5 salts (G398, PhytoTech, USA), 10 g/l sucrose, pH 5.7, 8 g/l agar, 160 mg/l Timentin, 1 mg/l IBA, 2 mg/l AgNO3, and 25 mg/l kanamycin.

## Supplementary Material

Web_Material_uhaf328

## Data Availability

Availability of Data and Materials. All the data necessary for evaluating the conclusions presented in the paper are included within the paper itself and/or the Supplementary Materials. All the materials produced during this study can be obtained from the corresponding author, T.B.S.
