# Supplementary material for: BrPIF4/BrBBX24-BrHB52-mediated hypocotyl modularity unlocks mechanized harvesting potential in Brassica rapa
Source: Hortic Res. 2025 Dec 8;13(3):uhaf328. doi: 10.1093/hr/uhaf328 (PMC12981329; doi:10.1093/hr/uhaf328)
Supplement: Web_Material_uhaf328 [file web_material_uhaf328.zip › Supplementary.docx]

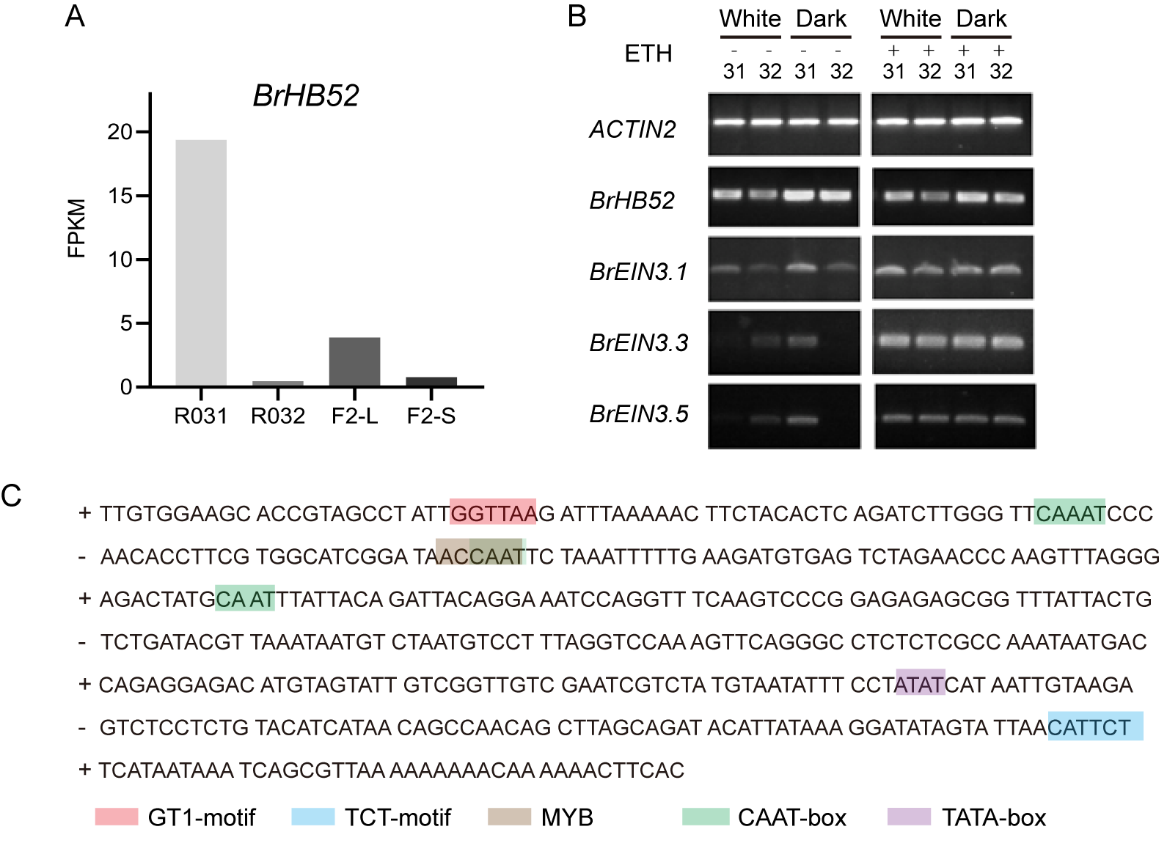


**Figure S1. The FPKM of *BrHB52* and the expression pattern of *BrHB52* under ethylene.**

1. The FPKM of *BrHB52* in R031L, R032S, the hypocotyls of long-hypocotyl (F2-L) and short-hypocotyl (F2-S) plants within the F2 population derived from the cross between R031L and R032S. B. Semi-quantitative PCR analysis of *BrHB52*, *BrHB5*2, *BrEIN3.1*, *BrEIN3.3* and *BrEIN3.5* expression under ETH treatment, with *ACTIN2* as the reference gene. Plant R031L and R032S on 1/2 MS medium supplemented with 5μM ethephon (ETH) or sterile water (negative control). After growing in darkness and white light for 7 days, respectively, observe the hypocotyl length and extract total RNA. C. The 251-bp sequence specifically inserted into the *BrHB52* promoter from R031L and its included cis-acting elements.

**
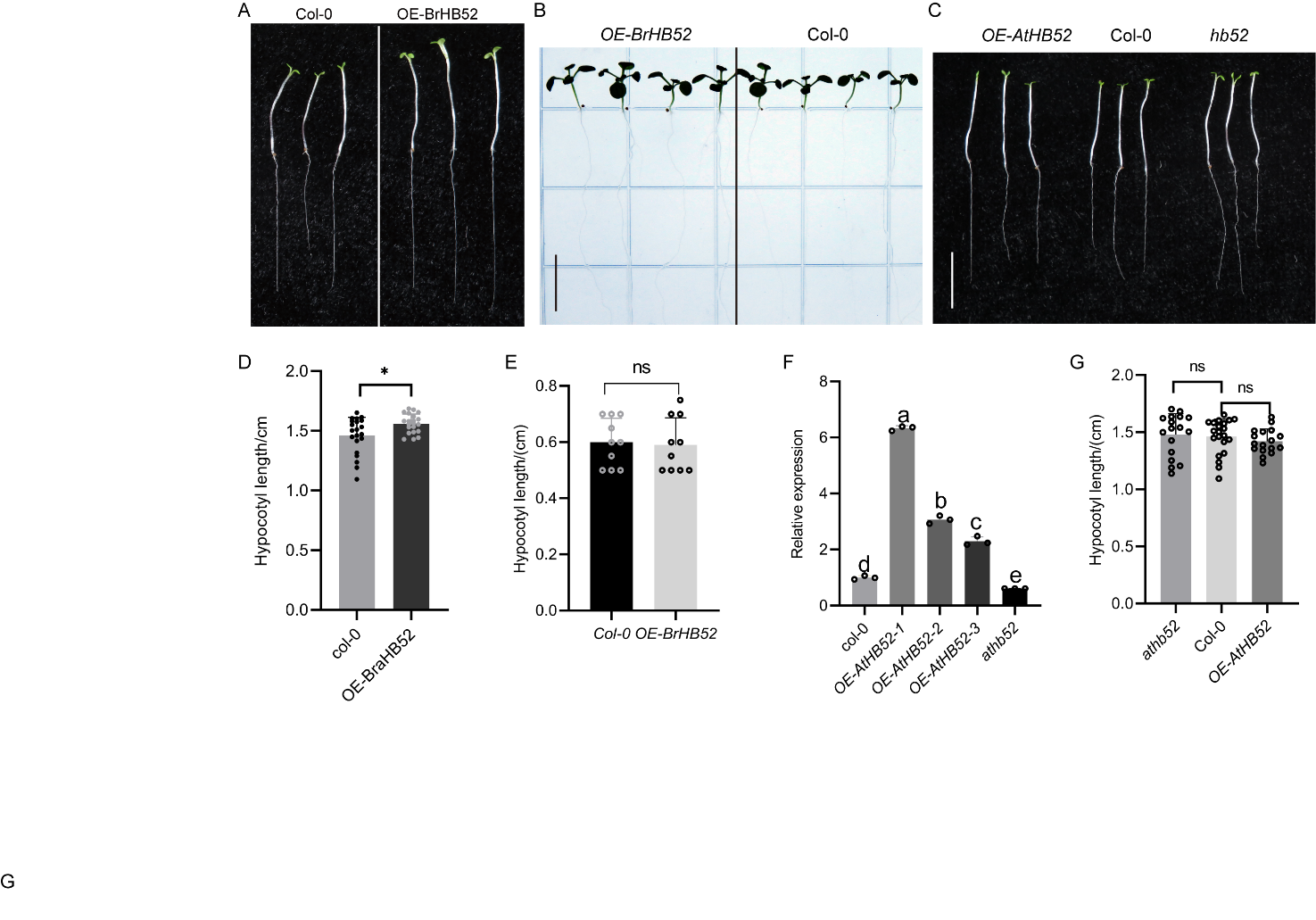
**

**Figure S2. The hypocotyl phenotypes of *BrHB52* transgenic plants.**

1. *BrHB52* overexpression in Col-0 increased hypocotyl length grown for 9 days on 1/2 MS medium under white light with intensity of 20 μmolm-^2^s^-1^in the incubator under short-day conditions. The scale bars in the relevant image indicate a length of 1 cm. B. Arabidopsis thaliana Columbia-0 (Col-0), transgenic plants *OE-BrHB52* grown for 15 days on 1/2 MS medium under white light with intensity of 50 μmolm-2s-1 under long-day conditions.in the incubator. Scale bars represent 1 cm. C. The mutations and overexpression of *ATHB52* did not affect the length of the hypocotyl. The scale bars in the relevant image indicate a length of 1 cm. D. Statistical results of hypocotyl length in Col-0 and transgenic Arabidopsis *OE-BrHB52* of A. Asterisks are used to denote significant disparities between Col-0 and the transgenic plants (**p*<0.01, Student’s *t*-test). E. Statistical results of hypocotyl length in Col-0 and transgenic Arabidopsis *OE-BrHB52* of B. Ns indicate no significant differences between Col-0 and transgenic plants (Student’s *t*-test). F. The expression of *AtHB52* in Col-0, *hb52* and *OE-AtHB52.* Identical letters signify non-significant disparities, while distinct letters indicate significant differences (*p* < 0.05, two-way ANOVA). G. Statistical results of hypocotyl length in Col-0, *hb52* and *OE-AtHB52.* Ns indicate no significant differences between Col-0 and transgenic plants (Student’s *t*-test).


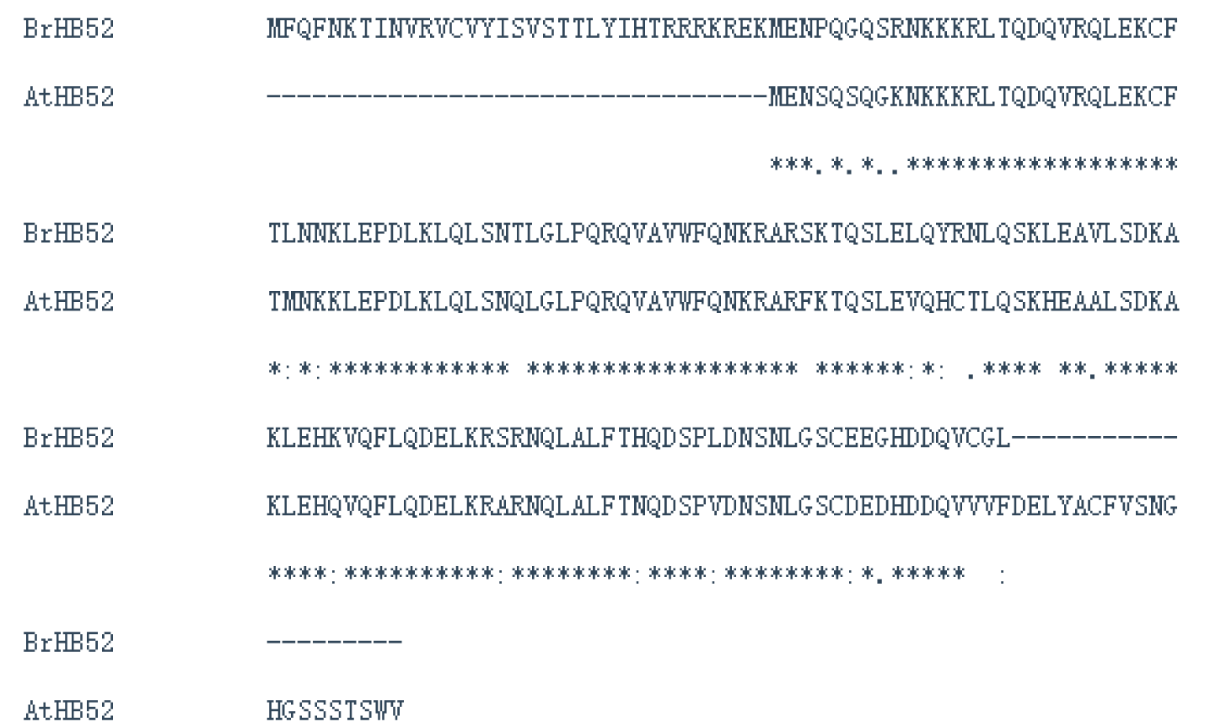
**Figure S3. Amino acid sequence alignment of BrHB52 and AtHB52**
