# Supplementary figures and images for: BrPIF4/BrBBX24-BrHB52-mediated hypocotyl modularity unlocks mechanized harvesting potential in Brassica rapa
Source: Hortic Res. 2025 Dec 8;13(3):uhaf328. doi: 10.1093/hr/uhaf328 (PMC12981329; doi:10.1093/hr/uhaf328)

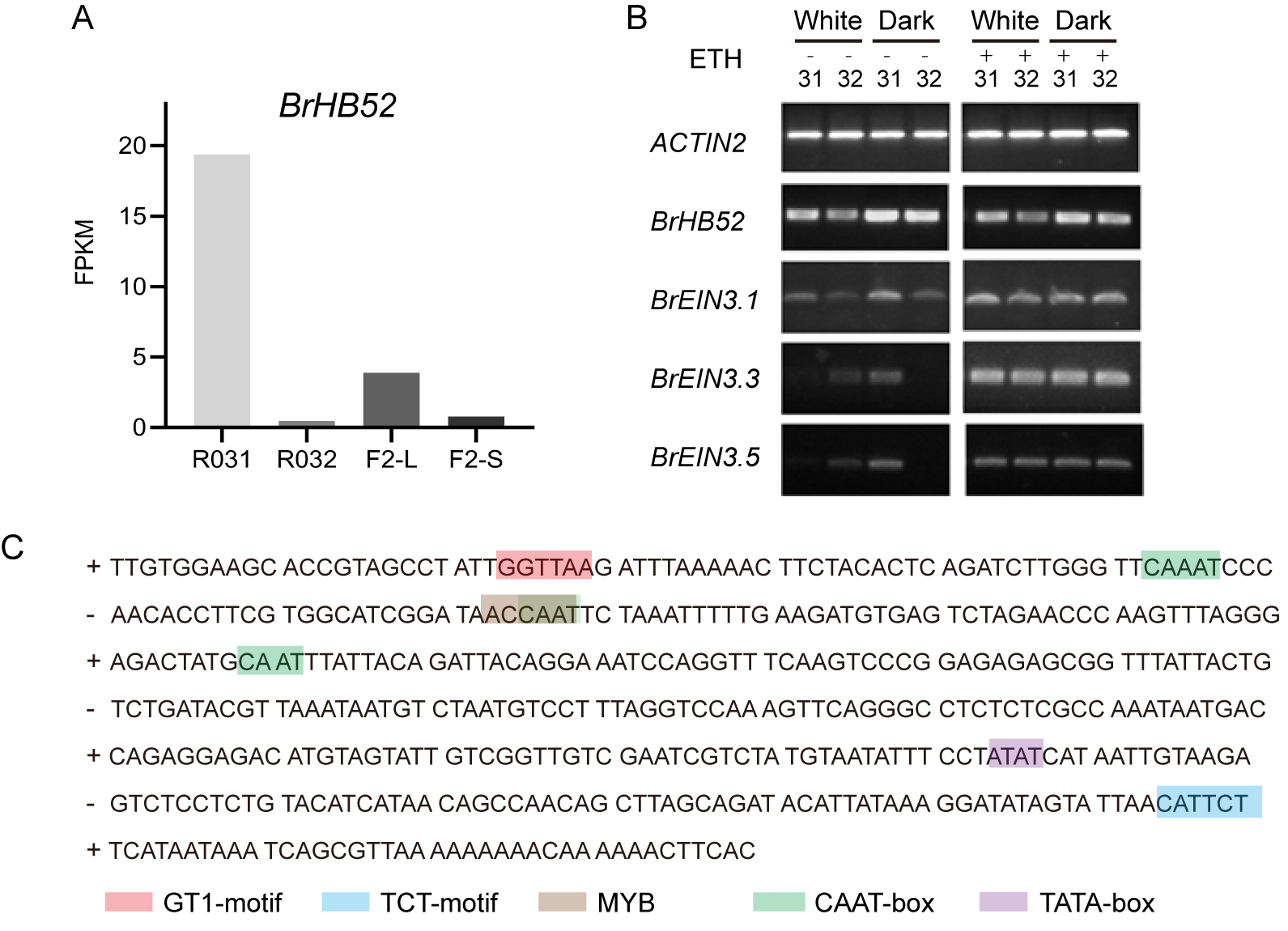

Supplement: Web_Material_uhaf328 [file web_material_uhaf328.zip › FIgureS1.tif]

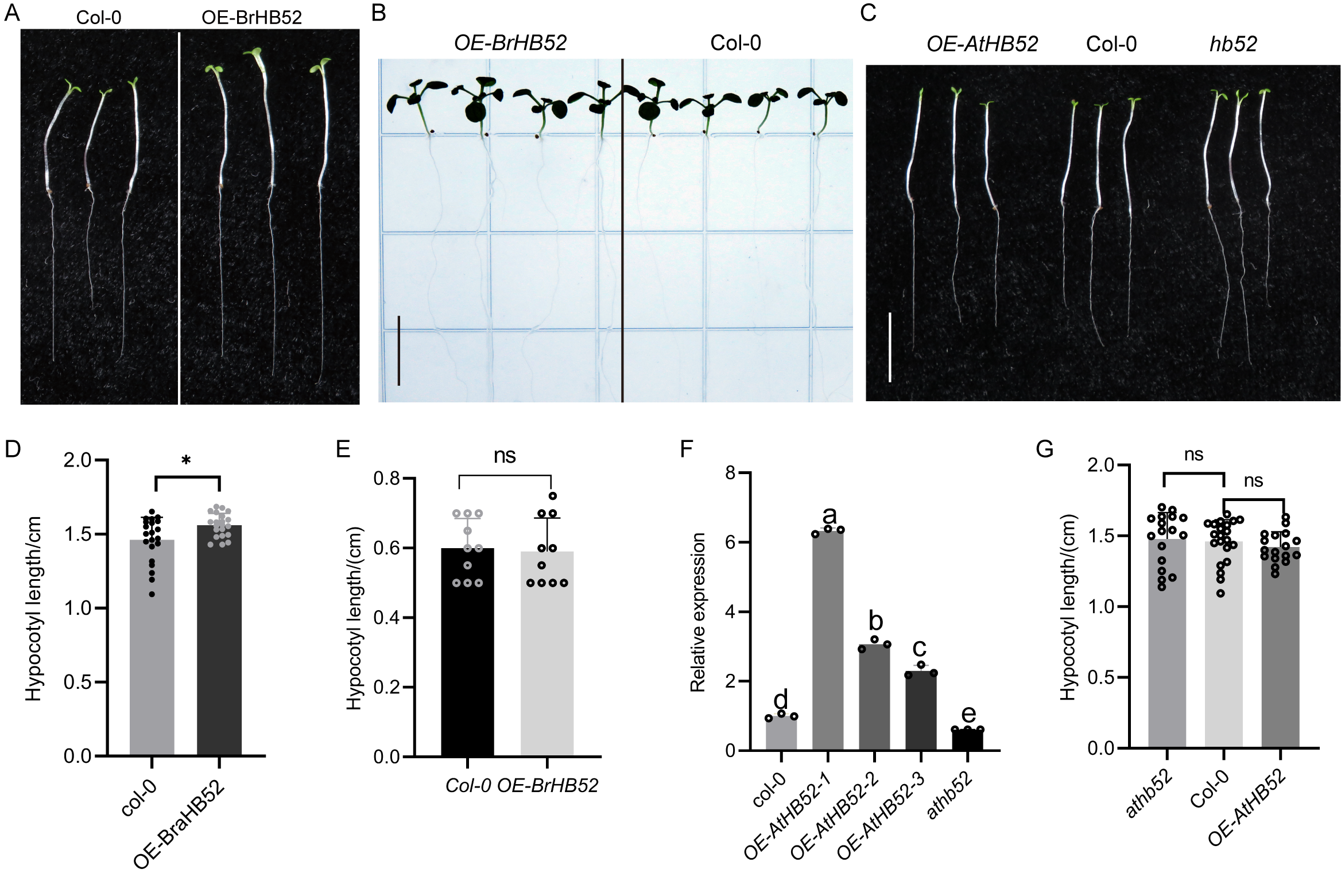

Supplement: Web_Material_uhaf328 [file web_material_uhaf328.zip › FIgureS2.tif]

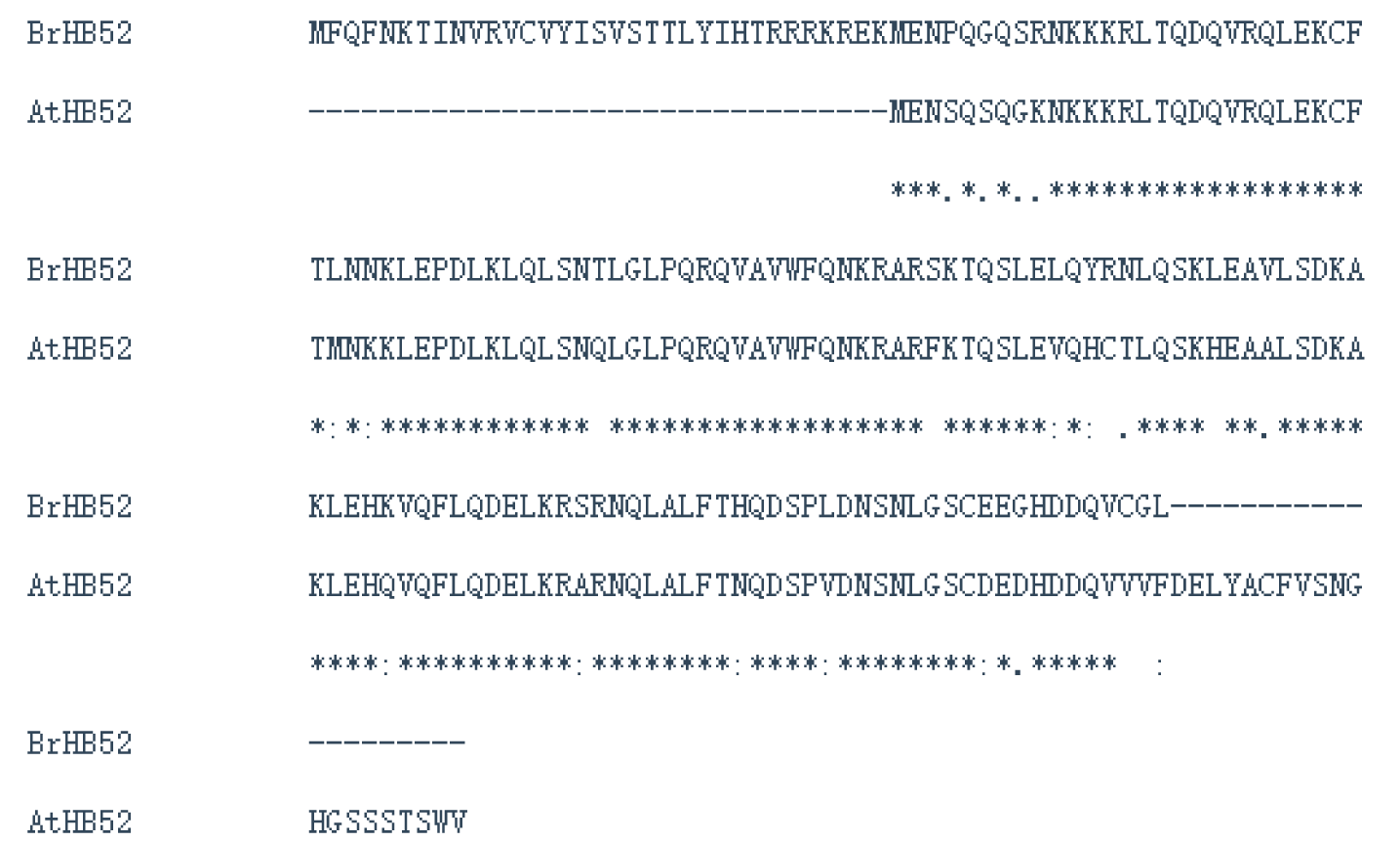

Supplement: Web_Material_uhaf328 [file web_material_uhaf328.zip › FigureS3.tif]
